# Supplementary material for: Comparative Brain Proteomic Analysis between Sham and Cerebral Ischemia Experimental Groups
Source: Int J Mol Sci. 2024 Jul 9;25(14):7538. doi: 10.3390/ijms25147538 (PMC11277324; doi:10.3390/ijms25147538)

Supplemental Table S1: Common proteins between the sham and ischemic groups identified by qualitative protein analysis. Only proteins with a false discovery rate <1% were selected.

UnitProt Code	Gene name	Protein Name
Q4KM93	TM177	Transmembrane protein 177
Q8K4Q9	CXA8	Gap junction alpha-8 protein
O35162	HSP13	Heat shock 70 kDa protein 13
O88869	RASF9	Ras association domain-containing protein 9
P20156	VGF	Neurosecretory protein VGF
P22057	PTGDS	Prostaglandin-H2 D-isomerase
P51882	CD5	T-cell surface glycoprotein CD5
P54319	PLAP	Phospholipase A-2-activating protein
Q07936	ANXA2	Annexin A2
Q1HG60	PIF1	ATP-dependent DNA helicase PIF1
Q4R180	ORC3	Origin recognition complex subunit 3
Q56R16	IMA6	Importin subunit alpha-6
Q5M7A4	UBA5	Ubiquitin-like modifier-activating enzyme 5
Q5U318	PEA15	Astrocytic phosphoprotein PEA-15
Q63014	AKAP8	A-kinase anchor protein 8
Q63484	AKT3	RAC-gamma serine/threonine-protein kinase
Q641Y5	ATG7	Ubiquitin-like modifier-activating enzyme ATG7
Q9EPA0	DRP2	Dystrophin-related protein 2
Q9ESH6	GLRX1	Glutaredoxin-1
Q9JKU6	STRBP	Spermatid perinuclear RNA-binding protein
Q9Y5E4	PCDB5	Protocadherin beta-5

Supplemental Table S2: Dysregulated proteins Sham vs. Control. Proteins considered dysregulated are those with a p-value < 0.05 and Log2 Fold Change (FC) > 1.5 or <0.6.

UnitProt Code	Gene name	Protein name	p-value	Log 2 FC
Q9JLZ1	GLRX3	Glutaredoxin-3	0,0080	1,2789
Q4QRB4	TBB3	Tubulin beta-3 chain	<0,0001	1,1147
P15791	KCC2D	Calcium/calmodulin-dependent protein kinase type II subunit delta	0,0242	1,1061
P13638	AT1B2	Sodium/potassium-transporting ATPase subunit beta-2	0,0112	1,0458
Q04940	NEUG	Neurogranin	<0,0001	1,0090
P63055	PCP4	Calmodulin regulator protein PCP4	<0,0001	0,9656
Q6IRK9	CBPQ	Carboxypeptidase Q	<0,0001	0,9599
Q9QXU9	PCS1N	ProSAAS	<0,0001	0,9001
P04631	S100B	Protein S100-B	0,0027	0,8989
Q5HZV9	PP1R7	Protein phosphatase 1 regulatory subunit 7	<0,0001	0,8772
P19468	GSH1	Glutamate--cysteine ligase catalytic subunit	<0,0001	0,8586
P63018	HSP7C	Heat shock cognate 71 kDa protein	<0,0001	0,8169
P04550	PTMS	Parathymosin	<0,0001	0,8139
P62744	AP2S1	AP-2 complex subunit sigma	0,0389	0,7586
Q5BJS7	CPNE9	Copine-9	<0,0001	0,7448
Q6AYT5	ARMT1	Damage-control phosphatase ARMT1	0,0117	0,7425
Q7TP54	RIPR2	Rho family-interacting cell polarization regulator 2	<0,0001	0,7396
P13668	STMN1	Stathmin	<0,0001	0,7331
Q6P799	SYSC	Serine--tRNA ligase, cytoplasmic	<0,0001	0,7257
P01041	CYTB	Cystatin-B	0,0280	0,7257
Q4KM74	SC22B	Vesicle-trafficking protein SEC22b	<0,0001	0,7180
P83868	TEBP	Prostaglandin E synthase 3	0,0023	0,7149
Q6PEC1	TBCA	Tubulin-specific chaperone A	0,0088	0,7098
P61206	ARF3	ADP-ribosylation factor 3	0,0048	0,7095
Q6Q0N1	CNDP2	Cytosolic non-specific dipeptidase	<0,0001	0,7003
Q6P9T8	TBB4B	Tubulin beta-4B chain	<0,0001	0,6879
P06761	BIP	Endoplasmic reticulum chaperone BiP	<0,0001	0,6848
P0DP31	CALM3	Calmodulin-3	0,0065	0,6828
P11980	KPYM	Pyruvate kinase PKM	<0,0001	0,6798
Q64303	PAK2	Serine/threonine-protein kinase PAK 2	<0,0001	0,6792
O55012	PICAL	Phosphatidylinositol-binding clathrin assembly protein	<0,0001	0,6746
P85108	TBB2A	Tubulin beta-2A chain	<0,0001	0,6703
P02401	RLA2	60S acidic ribosomal protein P2	0,0450	0,6684
Q9Z0G8	WIPF3	WAS/WASL-interacting protein family member 3	0,0020	0,6680
Q63537	SYN2	Synapsin-2	<0,0001	0,6638
P11232	THIO	Thioredoxin	0,0015	0,6623
P47942	DPYL2	Dihydropyrimidinase-related protein 2	<0,0001	0,6612
O35952	GLO2	Hydroxyacylglutathione hydrolase, mitochondrial	<0,0001	0,6606
Q9ER24	ATX10	Ataxin-10	0,0180	0,6603

Q63754	SYUB	Beta-synuclein	<0,0001	0,6511
P07633	PCCB	Propionyl-CoA carboxylase beta chain, mitochondrial	0,0207	0,6438
P04764	ENOA	Alpha-enolase	<0,0001	0,6433
P11980-2	KPYM	Isoform M2 of Pyruvate kinase PKM	0,0250	0,6378
Q07009	CAN2	Calpain-2 catalytic subunit	<0,0001	0,6307
Q9JHU0	DPYL5	Dihydropyrimidinase-related protein 5	<0,0001	0,6277
O35303	DNM1L	Dynamin-1-like protein	<0,0001	0,6265
Q6P7B0	SYWC	Tryptophan--tRNA ligase, cytoplasmic	<0,0001	0,6190
P69897	TBB5	Tubulin beta-5 chain	<0,0001	0,6080
Q9Z2G8	NP1L1	Nucleosome assembly protein 1-like 1	0,0169	0,5993
O35567	PUR9	Bifunctional purine biosynthesis protein ATIC	<0,0001	0,5967
Q5XIC0	ECI2	Enoyl-CoA delta isomerase 2	0,0192	-0,7147
P63088	PP1G	Serine/threonine-protein phosphatase PP1-gamma catalytic subunit	<0,0001	-0,7250
P06686	AT1A2	Sodium/potassium-transporting ATPase subunit alpha-2	<0,0001	-0,7368
P39069	KAD1	Adenylate kinase isoenzyme 1	<0,0001	-0,7629
P12839	NFM	Neurofilament medium polypeptide	0,0039	-0,7697
P35439	NMDZ1	Glutamate receptor ionotropic, NMDA 1	0,0049	-0,7833
P32551	QCR2	Cytochrome b-c1 complex subunit 2, mitochondrial	0,0058	-0,7971
P20788	UCRI	Cytochrome b-c1 complex subunit Rieske, mitochondrial	0,0043	-0,7973
Q5PQN0	NCALD	Neurocalcin-delta	0,0235	-0,8020
P19234	NDUV2	NADH dehydrogenase [ubiquinone] flavoprotein 2, mitochondrial	0,0039	-0,8094
Q68FY0	QCR1	Cytochrome b-c1 complex subunit 1, mitochondrial	0,0179	-0,8142
P67779	PHB	Prohibitin	0,0026	-0,8148
Q5BK63	NDUA9	NADH dehydrogenase [ubiquinone] 1 alpha subcomplex subunit 9, mitochondrial	0,0018	-0,8159
Q63965	SFXN1	Sideroflexin-1	0,0102	-0,8238
Q63622	DLG2	Disks large homolog 2	<0,0001	-0,8288
Q5XFW8	SEC13	Protein SEC13 homolog	0,0011	-0,8470
P09626	ATP4A	Potassium-transporting ATPase alpha chain 1	<0,0001	-0,8497
P13086	SUCA	Succinate--CoA ligase [ADP/GDP-forming] subunit alpha, mitochondrial	<0,0001	-0,8522
Q8CFD0	SFXN5	Sideroflexin-5	0,0011	-0,8560
Q812E9-2	GPM6A	Isoform 2 of Neuronal membrane glycoprotein M6-a	<0,0001	-0,8910
P21913	SDHB	Succinate dehydrogenase [ubiquinone] iron-sulfur subunit, mitochondrial	0,0007	-0,9087
P48500	TPIS	Triosephosphate isomerase	0,0013	-0,9162
P61589	RHOA	Transforming protein RhoA	0,0012	-0,9183
P47875	CSRP1	Cysteine and glycine-rich protein 1	<0,0001	-0,9415
Q9ESW0	DDB1	DNA damage-binding protein 1	<0,0001	-0,9651
Q9R1Z0	VDAC3	Voltage-dependent anion-selective channel protein 3	<0,0001	-0,9687

P60892	PRPS1	Ribose-phosphate pyrophosphokinase 1	<0,0001	-0,9716
Q6QIX3	ZNT3	Zinc transporter 3	<0,0001	-0,9848
P11661	NU5M	NADH-ubiquinone oxidoreductase chain 5	0,0009	-0,9936
P16036	MPCP	Phosphate carrier protein, mitochondrial	<0,0001	-1,0008
P16884	NFH	Neurofilament heavy polypeptide	<0,0001	-1,0355
P81155	VDAC2	Voltage-dependent anion-selective channel protein 2	<0,0001	-1,0384
Q9ERQ6	CSPG5	Chondroitin sulfate proteoglycan 5	0,0122	-1,1130
Q63345	MOG	Myelin-oligodendrocyte glycoprotein	0,0006	-1,1404
P31399	ATP5H	ATP synthase subunit d, mitochondrial	0,0083	-1,1559
Q9Z2L0	VDAC1	Voltage-dependent anion-selective channel protein 1	<0,0001	-1,1696
B0BNE5	ESTD	S-formylglutathione hydrolase	<0,0001	-1,1728
P30904	MIF	Macrophage migration inhibitory factor	<0,0001	-1,1784
P02770	ALBU	Albumin	<0,0001	-1,2149
P97700	M2OM	Mitochondrial 2-oxoglutarate/malate carrier protein	<0,0001	-1,2596
Q9JJK1	GPM6B	Neuronal membrane glycoprotein M6-b	<0,0001	-1,3177
Q09073	ADT2	ADP/ATP translocase 2	<0,0001	-1,3865
P60203	MYPR	Myelin proteolipid protein	0,0024	-1,3985
P54311	GBB1	Guanine nucleotide-binding protein G(I)/G(S)/G(T) subunit beta-1	<0,0001	-1,4450
Q05962	ADT1	ADP/ATP translocase 1	<0,0001	-1,5373
P02091	HBB1	Hemoglobin subunit beta-1	0,0049	-1,5408
P01946	HBA	Hemoglobin subunit alpha-1/2	0,0043	-1,7744
P59649	FXYD7	FXYD domain-containing ion transport regulator 7	<0,0001	-2,5023

Supplemental Table S3: SWATH-MS areas of the main upregulated proteins (Sham vs. Control)

UPREGULATED PROTEINS				
**Low Expression**	**Control**		**Sham**	
**Gene name**	**SWATH-MS areas**	**SD**	**SWATH-MS areas**	**SD**
**GLRX3**	3921	1588	7102	1432
**KCC2D**	5518	3813	9893	3372
**NEUG**	5267	1084	10437	2177
**High Expression**				
**TBB3**	442946	118936	896615	284537
**AT1B2**	70309	40825	136434	81064

Supplemental Table S4: SWATH-MS areas of the main downregulated proteins (Sham vs. Control)

DOWNREGULATED PROTEINS				
**Low Expression**	**Control**		**Sham**	
**Gene name**	**SWATH-MS areas**	**SD**	**SWATH-MS areas**	**SD**
**PRPS1**	15414	1379	8872	2553
**ZNT3**	8046	750	4624	1147
**NU5M**	23235	6819	12047	3429
**CSPG5**	7360	3660	3750	1232
**ESTD**	12851	5074	6275	2205
**MIF**	12772	4657	8255	1843
**GPM6B**	44147	9585	18905	4815
**ADT2**	86298	14276	34698	8909
**FXYD7**	32159	17879	7045	3985
**High Expression**				
**MPCP**	134862	34105	73094	18814
**NFH**	309069	41794	151148	47756
**VDAC2**	204110	56017	97023	31133
**MOG**	152697	56103	76897	19024
**ATP5H**	69947	10769	37527	17613
**VDAC1**	161728	30335	73247	15893
**ALBU**	199008	83883	99784	33275
**M2OM**	138990	28172	64160	20010
**MYPR**	6116828	2135216	2454250	798258
**GBB1**	147645	63639	53529	19005
**ADT1**	243357	47496	87711	22531
**HBB1**	114440	76649	56054	25687
**HBA**	395981	297864	158825	83849

Supplemental Table S5: Dysregulated proteins Ischemic vs. Control. Proteins considered dysregulated are those with a p-value < 0.05 and Log 2 Fold Change (FC) > 1.5 or <0.6.

UnitProt Code	Gene name	Protein name	p-value	Log 2 FC
P14480	FIBB	Fibrinogen beta chain	0,0274	3,2465
P42930	HSPB1	Heat shock protein beta-1	<0,0001	3,2242
P02770	ALBU	Albumin	<0,0001	2,9175
P0DMW1	HS71B	Heat shock 70 kDa protein 1B	<0,0001	2,4451
P17475	A1AT	Alpha-1-antiproteinase	<0,0001	2,1630
P01026	CO3	Complement C3	<0,0001	1,9008
P04276	VTDB	Vitamin D-binding protein	<0,0001	1,6907
P20059	HEMO	Hemopexin	0,0002	1,6022
P12346	TRFE	Serotransferrin	0,0002	1,5968
P10362	SCG2	Secretogranin-2	<0,0001	1,3582
P02091	HBB1	Hemoglobin subunit beta-1	0,0356	1,3367
P15791	KCC2D	Calcium/calmodulin-dependent protein kinase type II subunit delta	0,0237	1,3075
P01946	HBA	Hemoglobin subunit alpha-1/2	0,0487	1,2293
O08722	UNC5B	Netrin receptor UNC5B	0,0049	1,1630
P11980-2	KPYM	Isoform M2 of Pyruvate kinase PKM	<0,0001	1,1573
Q9QXU9	PCS1N	ProSAAS	<0,0001	1,1292
P14046	A1I3	Alpha-1-inhibitor 3	0,0003	1,1238
Q6AYT5	ARMT1	Damage-control phosphatase ARMT1	<0,0001	1,0638
P04764	ENOA	Alpha-enolase	<0,0001	1,0608
P24090	FETUA	Alpha-2-HS-glycoprotein	0,0002	1,0522
P48199	CRP	C-reactive protein	<0,0001	1,0333
P02401	RLA2	60S acidic ribosomal protein P2	0,0015	1,0307
Q04940	NEUG	Neurogranin	<0,0001	1,0058
P13668	STMN1	Stathmin	<0,0001	0,9606
Q6AXU6	JUPI1	Jupiter microtubule associated homolog 1	<0,0001	0,9556
P04550	PTMS	Parathymosin	<0,0001	0,9404
Q6P6V0	G6PI	Glucose-6-phosphate isomerase	<0,0001	0,9394
P62824	RAB3C	Ras-related protein Rab-3C	0,0121	0,9232
P32232	CBS	Cystathionine beta-synthase	0,0009	0,8844
A1L108	ARP5L	Actin-related protein 2/3 complex subunit 5-like protein	0,0080	0,8833
Q6PEC1	TBCA	Tubulin-specific chaperone A	5,05E-06	0,8763
P63055	PCP4	Calmodulin regulator protein PCP4	<0,0001	0,8646
Q5XIF4	SUMO3	Small ubiquitin-related modifier 3	<0,0001	0,8486
Q3MIE4	VAT1	Synaptic vesicle membrane protein VAT-1 homolog	<0,0001	0,8418
Q6IRK9	CBPQ	Carboxypeptidase Q	0,0007	0,8405
Q68FS4	AMPL	Cytosol aminopeptidase	<0,0001	0,8266
Q68FR8	TBA3	Tubulin alpha-3 chain	0,0070	0,8255
Q6PEC4	SKP1	S-phase kinase-associated protein 1	0,0035	0,7934
Q68FX1	MPI	Mannose-6-phosphate isomerase	0,0019	0,7867
Q99ML5	PCYOX	Prenylcysteine oxidase	0,0093	0,7842
O35567	PUR9	Bifunctional purine biosynthesis protein ATIC	<0,0001	0,7754

P10536	RAB1B	Ras-related protein Rab-1B	0,0031	0,7733
P70619	GSHR	Glutathione reductase (Fragment)	<0,0001	0,7407
Q6AYP5	CADM1	Cell adhesion molecule 1	0,0010	0,7040
P11232	THIO	Thioredoxin	0,0001	0,6902
P04785	PDIA1	Protein disulfide-isomerase	<0,0001	0,6877
P18298	METK2	S-adenosylmethionine synthase isoform type-2	<0,0001	0,6809
Q9ER24	ATX10	Ataxin-10	0,0146	0,6772
Q02253	MMSA	Methylmalonate-semialdehyde dehydrogenase [acylating], mitochondrial	0,0032	0,6701
P13676	ACPH	Acylamino-acid-releasing enzyme	0,0024	0,6675
Q6IMF3	K2C1	Keratin, type II cytoskeletal 1	0,0062	0,6645
P02625	PRVA	Parvalbumin alpha	0,0049	0,6570
P83868	TEBP	Prostaglandin E synthase 3	0,0079	0,6557
O08618	KPRB	Phosphoribosyl pyrophosphate synthase-associated protein 2	0,0021	0,6535
P62744	AP2S1	AP-2 complex subunit sigma	0,0139	0,6348
Q08163	CAP1	Adenylyl cyclase-associated protein 1	<0,0001	0,6327
Q9Z0G8	WIPF3	WAS/WASL-interacting protein family member 3	0,0024	0,6296
Q63041	A1M	Alpha-1-macroglobulin	0,0061	0,6252
P49088	ASNS	Asparagine synthetase [glutamine-hydrolyzing]	0,0196	0,6251
Q62658	FKB1A	Peptidyl-prolyl cis-trans isomerase FKBP1A	0,0001	0,6232
Q5XIG8	STRAP	Serine-threonine kinase receptor-associated protein	0,0068	0,6141
P11517	HBB2	Hemoglobin subunit beta-2	0,0123	0,6127
Q08602	PGTA	Geranylgeranyl transferase type-2 subunit alpha	0,0074	0,6100
Q4G017	NISCH	Nischarin	0,0203	0,6097
O08815	SLK	STE20-like serine/threonine-protein kinase	0,0095	0,6059
Q6P6Q2	K2C5	Keratin, type II cytoskeletal 5	0,0113	0,6053
P62278	RS13	40S ribosomal protein S13	0,0169	0,6023
Q5XIM5	CDV3	Protein CDV3 homolog	<0,0001	0,6022
Q9Z1A5	ULA1	NEDD8-activating enzyme E1 regulatory subunit	0,0012	0,5970
Q62950	DPYL1	Dihydropyrimidinase-related protein 1	<0,0001	0,5965
Q07205	IF5	Eukaryotic translation initiation factor 5	0,0015	0,5949
P50137	TKT	Transketolase	0,0001	0,5933
P14841	CYTC	Cystatin-C	<0,0001	0,5904
Q63610-3	TPM3	Isoform 3 of Tropomyosin alpha-3 chain	0,0167	0,5899
Q9WTT6	GUAD	Guanine deaminase	<0,0001	0,5877
Q5U2Z3	NP1L4	Nucleosome assembly protein 1-like 4	0,0160	0,5862
P52481	CAP2	Adenylyl cyclase-associated protein 2	<0,0001	0,5853
P41123	RL13	60S ribosomal protein L13	<0,0001	-0,7163
P13086	SUCA	Succinate--CoA ligase [ADP/GDP-forming] subunit alpha, mitochondrial	<0,0001	-0,7179
Q9JJK1	GPM6B	Neuronal membrane glycoprotein M6-b	0,0020	-0,7227
P46101-2	DPP6	Isoform DPPX-S of Dipeptidyl aminopeptidase-like protein 6	<0,0001	-0,7262
Q63092	CAMKV	CaM kinase-like vesicle-associated protein	<0,0001	-0,7275

P07340	AT1B1	Sodium/potassium-transporting ATPase subunit beta-1	<0,0001	-0,7286
P16036	MPCP	Phosphate carrier protein, mitochondrial	0,0028	-0,7319
Q8CFD0	SFXN5	Sideroflexin-5	0,0051	-0,7551
P06686	AT1A2	Sodium/potassium-transporting ATPase subunit alpha-2	<0,0001	-0,7634
P19511	AT5F1	ATP synthase F(0) complex subunit B1, mitochondrial	0,0004	-0,7655
P18508	GBRG2	Gamma-aminobutyric acid receptor subunit gamma-2	<0,0001	-0,7673
P11275	KCC2A	Calcium/calmodulin-dependent protein kinase type II subunit alpha	<0,0001	-0,7740
P11505	AT2B1	Plasma membrane calcium-transporting ATPase 1	0,0006	-0,7743
P35439	NMDZ1	Glutamate receptor ionotropic, NMDA 1	0,0060	-0,7763
P62804	H4	Histone H4	<0,0001	-0,7777
P68403-2	KPCB	Isoform Beta-II of Protein kinase C beta type	<0,0001	-0,7812
Q9QXY2	SRCN1	SRC kinase signaling inhibitor 1	<0,0001	-0,7823
P07722-2	MAG	Isoform S-MAG of Myelin-associated glycoprotein	0,0026	-0,7830
P09951	SYN1	Synapsin-1	<0,0001	-0,7839
O35867	NEB1	Neurabin-1	<0,0001	-0,7873
P49621	DGKB	Diacylglycerol kinase beta	0,0130	-0,7906
Q1AAU6	ASAP1	Arf-GAP with SH3 domain, ANK repeat and PH domain-containing protein 1	<0,0001	-0,7909
Q9QUH6-4	SYGP1	Isoform 4 of Ras/Rap GTPase-activating protein SynGAP	<0,0001	-0,7933
P59215	GNAO	Guanine nucleotide-binding protein G(o) subunit alpha	<0,0001	-0,7954
Q9JLU4	SHAN3	SH3 and multiple ankyrin repeat domains protein 3	0,0015	-0,8044
Q561S0	NDUAA	NADH dehydrogenase [ubiquinone] 1 alpha subcomplex subunit 10, mitochondrial	0,0002	-0,8069
Q8CGU4	AGAP2	Arf-GAP with GTPase, ANK repeat and PH domain-containing protein 2	<0,0001	-0,8081
Q3KR86	MIC60	MIC	0,0060	-0,8176
Q9WV48	SHAN1	SH3 and multiple ankyrin repeat domains protein 1	0,0047	-0,8212
Q5BK63	NDUA9	NADH dehydrogenase [ubiquinone] 1 alpha subcomplex subunit 9, mitochondrial	0,0016	-0,8217
P24942	EAA1	Excitatory amino acid transporter 1	<0,0001	-0,8222
Q6QIX3	ZNT3	Zinc transporter 3	0,0054	-0,8232
P20788	UCRI	Cytochrome b-c1 complex subunit Rieske, mitochondrial	0,0021	-0,8248
Q00715	H2B1	Histone H2B type 1	<0,0001	-0,8284
P11951	CX6C2	Cytochrome c oxidase subunit 6C-2	0,0108	-0,8288
Q9R1Z0	VDAC3	Voltage-dependent anion-selective channel protein 3	<0,0001	-0,8306
P62813	GBRA1	Gamma-aminobutyric acid receptor subunit alpha-1	<0,0001	-0,8352
P19234	NDUV2	NADH dehydrogenase [ubiquinone] flavoprotein 2, mitochondrial	0,0007	-0,8397
Q5XFW8	SEC13	Protein SEC13 homolog	0,0009	-0,8407

Q66HF1	NDUS1	NADH-ubiquinone oxidoreductase 75 kDa subunit, mitochondrial	0,0021	-0,8410
Q63622	DLG2	Disks large homolog 2	<0,0001	-0,8416
Q9ERQ6	CSPG5	Chondroitin sulfate proteoglycan 5	0,0403	-0,8419
P27952	RS2	40S ribosomal protein S2	0,0045	-0,8421
P06687	AT1A3	Sodium/potassium-transporting ATPase subunit alpha-3	<0,0001	-0,8463
P31596	EAA2	Excitatory amino acid transporter 2	<0,0001	-0,8476
D3ZAF6	ATPK	ATP synthase subunit f, mitochondrial	0,0202	-0,8668
P00406	COX2	Cytochrome c oxidase subunit 2	0,0032	-0,8720
Q63965	SFXN1	Sideroflexin-1	0,0010	-0,8751
A0A0G2JUG7	IQEC1	IQ motif and SEC7 domain-containing protein 1	<0,0001	-0,8786
P31399	ATP5H	ATP synthase subunit d, mitochondrial	0,0065	-0,8893
P11661	NU5M	NADH-ubiquinone oxidoreductase chain 5	0,0026	-0,8915
Q7TQ16	QCR8	Cytochrome b-c1 complex subunit 8	0,0179	-0,8936
Q64542-2	AT2B4	Isoform XA of Plasma membrane calcium-transporting ATPase 4	<0,0001	-0,9050
P09626	ATP4A	Potassium-transporting ATPase alpha chain 1	0,0002	-0,9086
Q5XIH7	PHB2	Prohibitin-2	0,0016	-0,9090
O08679	MARK2	Serine/threonine-protein kinase MARK2	0,0002	-0,9124
P47819	GFAP	Glial fibrillary acidic protein	0,0034	-0,9439
P60892	PRPS1	Ribose-phosphate pyrophosphokinase 1	<0,0001	-0,9492
P01830	THY1	Thy-1 membrane glycoprotein	<0,0001	-0,9562
P21913	SDHB	Succinate dehydrogenase [ubiquinone] iron-sulfur subunit, mitochondrial	0,0002	-0,9593
Q63633	S12A5	Solute carrier family 12 member 5	<0,0001	-0,9696
Q68FY0	QCR1	Cytochrome b-c1 complex subunit 1, mitochondrial	0,0006	-0,9825
P08413	KCC2B	Calcium/calmodulin-dependent protein kinase type II subunit beta	<0,0001	-0,9907
P32551	QCR2	Cytochrome b-c1 complex subunit 2, mitochondrial	0,0004	-1,0262
P54311	GBB1	Guanine nucleotide-binding protein G(I)/G(S)/G(T) subunit beta-1	<0,0001	-1,0272
P67779	PHB	Prohibitin	0,0001	-1,0294
Q09073	ADT2	ADP/ATP translocase 2	0,0001	-1,0828
P13638	AT1B2	Sodium/potassium-transporting ATPase subunit beta-2	0,0104	-1,0829
Q9Z327-3	SYNPO	Isoform 3 of Synaptopodin	<0,0001	-1,0856
D3ZBN0	H15	Histone H1.5	0,0106	-1,1101
Q812E9-2	GPM6A	Isoform 2 of Neuronal membrane glycoprotein M6-a	<0,0001	-1,1145
P15999	ATPA	ATP synthase subunit alpha, mitochondrial	0,0018	-1,1294
Q9Z1T4	CNKR2	Connector enhancer of kinase suppressor of ras 2	<0,0001	-1,1366
P15865	H14	Histone H1.4	<0,0001	-1,1700
P97700	M2OM	Mitochondrial 2-oxoglutarate/malate carrier protein	<0,0001	-1,2016
P10719	ATPB	ATP synthase subunit beta, mitochondrial	0,0012	-1,2331
P02688	MBP	Myelin basic protein	0,0002	-1,2422

Q9Z2L0	VDAC1	Voltage-dependent anion-selective channel protein 1	<0,0001	-1,2864
Q05962	ADT1	ADP/ATP translocase 1	0,0002	-1,2953
Q63345	MOG	Myelin-oligodendrocyte glycoprotein	<0,0001	-1,3464
P19527	NFL	Neurofilament light polypeptide	<0,0001	-1,3471
Q06647	ATPO	ATP synthase subunit	0,0004	-1,3766
Q9ESQ7	PSD1	PH and SEC7 domain-containing protein 1	<0,0001	-1,3783
P81155	VDAC2	Voltage-dependent anion-selective channel protein 2	<0,0001	-1,3799
P08050	CXA1	Gap junction alpha-1 protein	0,0133	-1,4020
P23565	AINX	Alpha-internexin	<0,0001	-1,4548
P16884	NFH	Neurofilament heavy polypeptide	<0,0001	-1,4848
P60203	MYPR	Myelin proteolipid protein	0,0006	-1,6144
P97834	CSN1	COP9 signalosome complex subunit 1	<0,0001	-1,6400
P43278	H10	Histone H1.0	<0,0001	-1,6411
P12839	NFM	Neurofilament medium polypeptide	<0,0001	-1,6417
Q99P82	CLD11	Claudin-11	0,0004	-1,6532
Q6LED0	H31	Histone H3.1	<0,0001	-1,8348
P59649	FXYD7	FXYD domain-containing ion transport regulator 7	<0,0001	-2,0239

Supplemental Table S6: SWATH-MS areas of the main upregulated proteins (Ischemia vs. Control).

**UPREGULATED PROTEINS**				
**Low Expression**	**Control**		**Ischemia**	
**Gene name**	**SWATH-MS areas**	**SD**	**SWATH-MS areas**	**SD**
**FIBB**	6187	1731	31997	28556
**HSPB1**	1196	285	7292	3483
**CO3**	11385	1326	27227	5792
**VTDB**	4878	1849	9320	2081
**SCG2**	13711	2330	33490	8210
**KCC2D**	5518	3813	5518	4370
**UNC5B**	5763	1878	5763	2643
**ARMT1**	1504	605	1504	274
**CRP**	4834	1976	6522	1040
**RLA2**	7895	3701	14900	3150
**NEUG**	5267	1084	9057	2391
**FETUA**	27211	6847	39415	8902
**High Expression**				
**ALBU**	199008	83883	697256	187893
**HS71B**	43310	2582	144072	42590
**A1AT**	34880	2587	96451	26553
**HEMO**	24365	4282	52206	11915
**TRFE**	66560	9331	131608	24383
**HBB1**	114440	76649	173965	111153
**HBA**	395981	297864	555116	385974
**KPYM**	359284	69795	589084	98481

**PCS1N**	37064	17923	60338	7080
**A1I3**	70109	12564	113890	15470
**ENOA**	969659	137549	1367233	299615

Supplemental Table S7: SWATH-MS areas of the main downregulated proteins (Ischemia vs. Control).

**DOWNREGULATED PROTEINS**				
**Low Expression**	**Control**		**Ischemia**	
**Gene name**	**SWATH-MS areas**	**SD**	**SWATH-MS areas**	**SD**
**H15**	8303	3786	4951	724
**CNKR2**	7416	990	4607	869
**PSD11**	6172	1106	8181	1112
**H10**	27464	5709	10474	2637
**CLD11**	31614	6247	11071	1563
**H31**	72607	23625	5518	5763
**FXYD7**	32159	17879	5763	562
**High Expression**				
**QCR1**	314078	101175	151887	32152
**KCC2B**	91453	24165	53044	6308
**QCR2**	153203	45248	74271	16701
**GBB1**	147645	63639	70507	11485
**PHB**	96093	28523	44950	7768
**ADT2**	86298	14276	38365	10103
**AT1B2**	70309	40825	39931	11262
**SYNPO**	223134	33367	125017	19574
**GPM6A**	197332	47123	90834	8365
**ATPA**	890979	328016	494807	103654
**H14**	301092	77852	139689	26155
**M2OM**	138990	28172	55761	8911
**ATPB**	1008718	376945	550027	70451
**MBP**	603899	169202	370715	58099
**VDAC1**	161728	30335	71669	9873
**ADT1**	243357	47496	103982	23076
**MOG**	152697	56103	61183	2995
**NFL**	480921	102451	191586	20847
**ATPO**	260277	61914	107858	25592
**VDAC2**	204110	56017	76441	14740
**CXA1**	152607	49425	80318	19477
**AINX**	950085	219298	363717	53300
**NFH**	309069	41794	116623	10620
**MYPR**	6116828	2135216	1835242	380783
**CSN1**	254321	61135	120736	16318
**NFM**	226361	50582	80643	17233

Supplemental Table S8: Common dysregulated proteins in the sham group. Proteins considered dysregulated are those with a p-value < 0.05 and Log 2Fold Change (FC) > 1.5 or <0.6.

UnitProt Code	Gene name	Protein name	p-value	Log2 (FC)
P15791	KCC2D	Calcium/calmodulin-dependent protein kinase type II subunit delta	0,0242	1,1061
P13638	AT1B2	Sodium/potassium-transporting ATPase subunit beta-2	0,0112	1,0458
Q04940	NEUG	Neurogranin	<0,0001	1,0090
P63055	PCP4	Calmodulin regulator protein PCP4	<0,0001	0,9656
Q6IRK9	CBPQ	Carboxypeptidase Q	<0,0001	0,9599
Q9QXU9	PCS1N	ProSAAS	<0,0001	0,9001
P04550	PTMS	Parathymosin	<0,0001	0,8139
P62744	AP2S1	AP-2 complex subunit sigma	0,0389	0,7586
Q6AYT5	ARMT1	Damage-control phosphatase ARMT1	0,0117	0,7425
P13668	STMN1	Stathmin	<0,0001	0,7331
P83868	TEBP	Prostaglandin E synthase 3	0,0023	0,7149
Q6PEC1	TBCA	Tubulin-specific chaperone A	0,0088	0,7098
P11980	KPYM	Pyruvate kinase PKM	<0,0001	0,6798
P02401	RLA2	60S acidic ribosomal protein P2	0,0450	0,6684
Q9Z0G8	WIPF3	WAS/WASL-interacting protein family member 3	0,0020	0,6680
P11232	THIO	Thioredoxin	0,0015	0,6623
Q9ER24	ATX10	Ataxin-10	0,0180	0,6603
P04764	ENOA	Alpha-enolase	<0,0001	0,6433
O35567	PUR9	Bifunctional purine biosynthesis protein ATIC	<0,0001	0,5967
P06686	AT1A2	Sodium/potassium-transporting ATPase subunit alpha-2	<0,0001	-0,7368
P12839	NFM	Neurofilament medium polypeptide	0,0039	-0,7697
P35439	NMDZ1	Glutamate receptor ionotropic, NMDA 1	0,0049	-0,7833
P32551	QCR2	Cytochrome b-c1 complex subunit 2, mitochondrial	0,0058	-0,7971
P20788	UCRI	Cytochrome b-c1 complex subunit Rieske, mitochondrial	0,0043	-0,7973
P19234	NDUV2	NADH dehydrogenase [ubiquinone] flavoprotein 2, mitochondrial	0,0039	-0,8094
Q68FY0	QCR1	Cytochrome b-c1 complex subunit 1, mitochondrial	0,0179	-0,8142
P67779	PHB	Prohibitin	0,0026	-0,8148
Q5BK63	NDUA9	NADH dehydrogenase [ubiquinone] 1 alpha subcomplex subunit 9, mitochondrial	0,0018	-0,8159
Q63965	SFXN1	Sideroflexin-1	0,0102	-0,8238
Q63622	DLG2	Disks large homolog 2	<0,0001	-0,8288
Q5XFW8	SEC13	Protein SEC13 homolog	0,0011	-0,8470
P09626	ATP4A	Potassium-transporting ATPase alpha chain 1	<0,0001	-0,8497
P13086	SUCA	Succinate--CoA ligase [ADP/GDP-forming] subunit alpha, mitochondrial	<0,0001	-0,8522
Q8CFD0	SFXN5	Sideroflexin-5	0,0011	-0,8560
Q812E9-2	GPM6A	Isoform 2 of Neuronal membrane glycoprotein M6-a	<0,0001	-0,8910

P21913	SDHB	Succinate dehydrogenase [ubiquinone] iron-sulfur subunit, mitochondrial	0,0007	-0,9087
Q9R1Z0	VDAC3	Voltage-dependent anion-selective channel protein 3	<0,0001	-0,9687
P60892	PRPS1	Ribose-phosphate pyrophosphokinase 1	<0,0001	-0,9716
Q6QIX3	ZNT3	Zinc transporter 3	<0,0001	-0,9848
P11661	NU5M	NADH-ubiquinone oxidoreductase chain 5	0,0009	-0,9936
P16036	MPCP	Phosphate carrier protein, mitochondrial	<0,0001	-1,0008
P16884	NFH	Neurofilament heavy polypeptide	<0,0001	-1,0355
P81155	VDAC2	Voltage-dependent anion-selective channel protein 2	<0,0001	-1,0384
Q9ERQ6	CSPG5	Chondroitin sulfate proteoglycan 5	0,0122	-1,1130
Q63345	MOG	Myelin-oligodendrocyte glycoprotein	0,0006	-1,1404
P31399	ATP5H	ATP synthase subunit d, mitochondrial	0,0083	-1,1559
Q9Z2L0	VDAC1	Voltage-dependent anion-selective channel protein 1	<0,0001	-1,1696
P02770	ALBU	Albumin	<0,0001	-1,2149
P97700	M2OM	Mitochondrial 2-oxoglutarate/malate carrier protein	<0,0001	-1,2596
Q9JJK1	GPM6B	Neuronal membrane glycoprotein M6-b	<0,0001	-1,3177
Q09073	ADT2	ADP/ATP translocase 2	<0,0001	-1,3865
P60203	MYPR	Myelin proteolipid protein	0,0024	-1,3985
P54311	GBB1	Guanine nucleotide-binding protein G(I)/G(S)/G(T) subunit beta-1	<0,0001	-1,4450
Q05962	ADT1	ADP/ATP translocase 1	<0,0001	-1,5373
P02091	HBB1	Hemoglobin subunit beta-1	0,0049	-1,5408
P01946	HBA	Hemoglobin subunit alpha-1/2	0,0043	-1,7744
P59649	FXYD7	FXYD domain-containing ion transport regulator 7	<0,0001	-2,5023

Supplemental Table S9: Common dysregulated proteins in the ischemic group. Proteins considered dysregulated are those with a p-value < 0.05 and a Log 2 Fold Change (FC) > 1.5 or <0.6.

UnitProt Code	Gene name	Protein name	p-value	Log 2 FC
P02770	ALBU	Albumin	<0,0001	2,9175
P02091	HBB1	Hemoglobin subunit beta-1	0,0356	1,3367
P15791	KCC2D	Calcium/calmodulin-dependent protein kinase type II subunit delta	0,0237	1,3075
P01946	HBA	Hemoglobin subunit alpha-1/2	0,0487	1,2293
P11980-2	KPYM	Isoform M2 of Pyruvate kinase PKM	<0,0001	1,1573
Q9QXU9	PCS1N	ProSAAS	<0,0001	1,1292
Q6AYT5	ARMT1	Damage-control phosphatase ARMT1	<0,0001	1,0638
P04764	ENOA	Alpha-enolase	<0,0001	1,0608
P02401	RLA2	60S acidic ribosomal protein P2	0,0015	1,0307
Q04940	NEUG	Neurogranin	<0,0001	1,0058
P13668	STMN1	Stathmin	<0,0001	0,9606
P04550	PTMS	Parathymosin	<0,0001	0,9404
Q6PEC1	TBCA	Tubulin-specific chaperone A	<0,0001	0,8763
P63055	PCP4	Calmodulin regulator protein PCP4	<0,0001	0,8646
Q6IRK9	CBPQ	Carboxypeptidase Q	0,0007	0,8405
O35567	PUR9	Bifunctional purine biosynthesis protein ATIC	<0,0001	0,7754
P11232	THIO	Thioredoxin	0,0001	0,6902
Q9ER24	ATX10	Ataxin-10	0,0146	0,6772
P83868	TEBP	Prostaglandin E synthase 3	0,0079	0,6557
P62744	AP2S1	AP-2 complex subunit sigma	0,0139	0,6348
Q9Z0G8	WIPF3	WAS/WASL-interacting protein family member 3	0,0024	0,6296
P13086	SUCA	Succinate--CoA ligase [ADP/GDP-forming] subunit alpha, mitochondrial	<0,0001	-0,7179
Q9JJK1	GPM6B	Neuronal membrane glycoprotein M6-b	0,0020	-0,7227
P16036	MPCP	Phosphate carrier protein, mitochondrial	0,0028	-0,7319
Q8CFD0	SFXN5	Sideroflexin-5	0,0051	-0,7551
P06686	AT1A2	Sodium/potassium-transporting ATPase subunit alpha-2	<0,0001	-0,7634
P35439	NMDZ1	Glutamate receptor ionotropic, NMDA 1	0,0060	-0,7763
Q5BK63	NDUA9	NADH dehydrogenase [ubiquinone] 1 alpha subcomplex subunit 9, mitochondrial	0,0016	-0,8217
Q6QIX3	ZNT3	Zinc transporter 3	0,0054	-0,8232
P20788	UCRI	Cytochrome b-c1 complex subunit Rieske, mitochondrial	0,0021	-0,8248
Q9R1Z0	VDAC3	Voltage-dependent anion-selective channel protein 3	<0,0001	-0,8306
P19234	NDUV2	NADH dehydrogenase [ubiquinone] flavoprotein 2, mitochondrial	0,0007	-0,8397
Q5XFW8	SEC13	Protein SEC13 homolog	0,0009	-0,8407
Q63622	DLG2	Disks large homolog 2	<0,0001	-0,8416
Q9ERQ6	CSPG5	Chondroitin sulfate proteoglycan 5	0,0403	-0,8419
Q63965	SFXN1	Sideroflexin-1	0,0010	-0,8751
P31399	ATP5H	ATP synthase subunit d, mitochondrial	0,0065	-0,8893

P11661	NU5M	NADH-ubiquinone oxidoreductase chain 5	0,0026	-0,8915
P09626	ATP4A	Potassium-transporting ATPase alpha chain 1	0,0002	-0,9086
P60892	PRPS1	Ribose-phosphate pyrophosphokinase 1	<0,0001	-0,9492
P21913	SDHB	Succinate dehydrogenase [ubiquinone] iron-sulfur subunit, mitochondrial	0,0002	-0,9593
Q68FY0	QCR1	Cytochrome b-c1 complex subunit 1, mitochondrial	0,0006	-0,9825
P32551	QCR2	Cytochrome b-c1 complex subunit 2, mitochondrial	0,0004	-1,0262
P54311	GBB1	Guanine nucleotide-binding protein G(I)/G(S)/G(T) subunit beta-1	<0,0001	-1,0272
P67779	PHB	Prohibitin	0,0001	-1,0294
Q09073	ADT2	ADP/ATP translocase 2	0,0001	-1,0828
P13638	AT1B2	Sodium/potassium-transporting ATPase subunit beta-2	0,0104	-1,0829
Q812E9-2	GPM6A	Isoform 2 of Neuronal membrane glycoprotein M6-a	<0,0001	-1,1145
P97700	M2OM	Mitochondrial 2-oxoglutarate/malate carrier protein	<0,0001	-1,2016
Q9Z2L0	VDAC1	Voltage-dependent anion-selective channel protein 1	<0,0001	-1,2864
Q05962	ADT1	ADP/ATP translocase 1	0,0002	-1,2953
Q63345	MOG	Myelin-oligodendrocyte glycoprotein	<0,0001	-1,3464
P81155	VDAC2	Voltage-dependent anion-selective channel protein 2	<0,0001	-1,3799
P16884	NFH	Neurofilament heavy polypeptide	<0,0001	-1,4848
P60203	MYPR	Myelin proteolipid protein	0,0006	-1,6144
P12839	NFM	Neurofilament medium polypeptide	<0,0001	-1,6417
P59649	FXYD7	FXYD domain-containing ion transport regulator 7	<0,0001	-2,0239

Supplemental Figure S1: Venn diagram showing the overlap of common dysregulated proteins identified by SWATH-MS, common proteins between sham and ischemia by DDA, and the protein pool common to control, sham and ischemic detected by DDA. Figures 5 (A) was created using FunRich program.

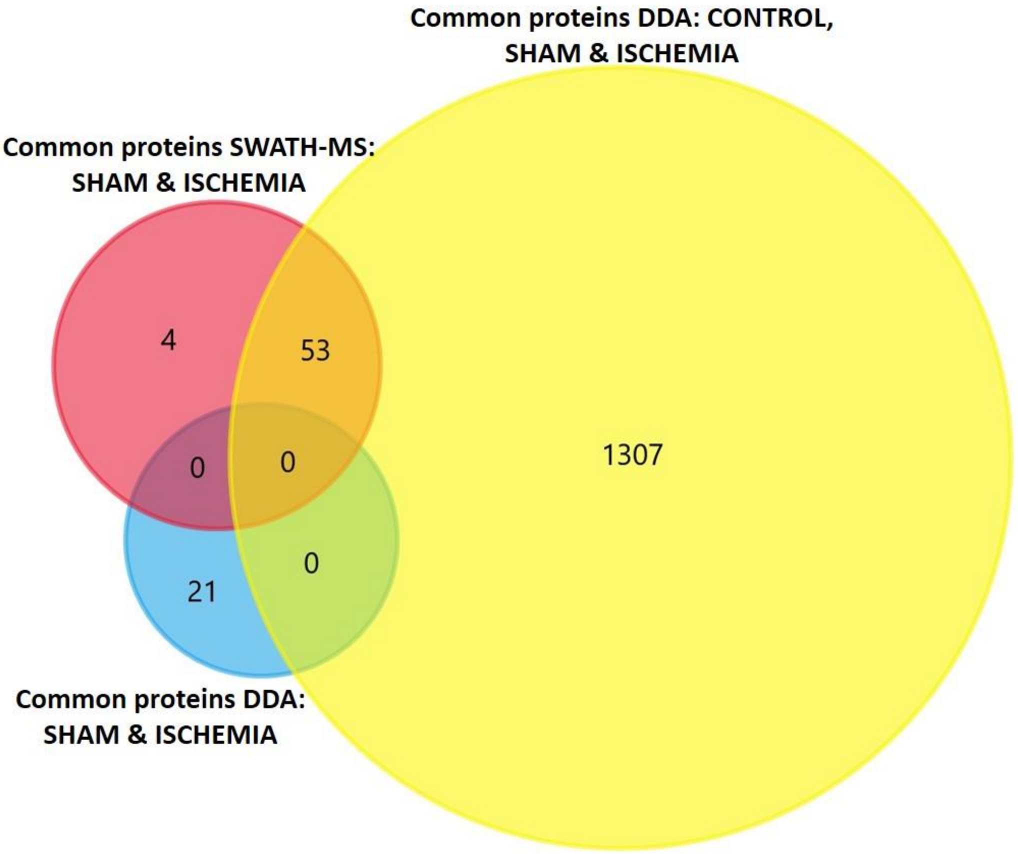

Supplemental Figure S2: Transient intraluminal middle cerebral artery occlusion (tMCAO) model representation. **A)** Schematic diagram of the rat carotid system. **B)** Contralateral artery location and isolation. **C**) Exposure and ligation of CCA, ECA, ICA and PPA. **D**) Occlusion of the MCA by means of the filament, which is introduced through the ECA and ICA. Common carotid artery (CCA). External carotid artery (ECA). Internal carotid artery (ICA). Pterygopalatine artery (PPA). Middle cerebral artery (MCA). Figure was created using BioRender.

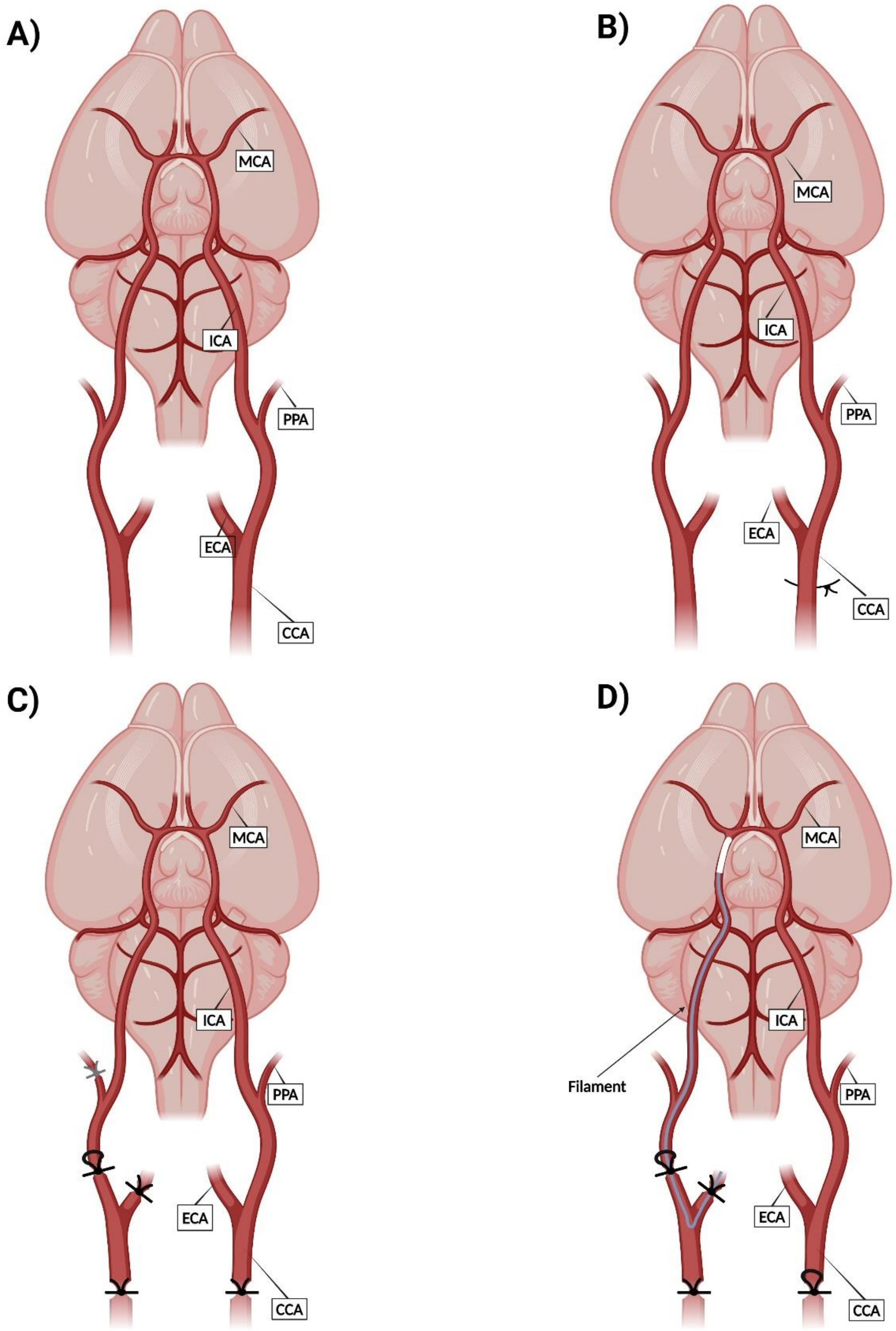

Supplemental Figure S3: Schematic representation of the experimental study. Figure was created using BioRender.

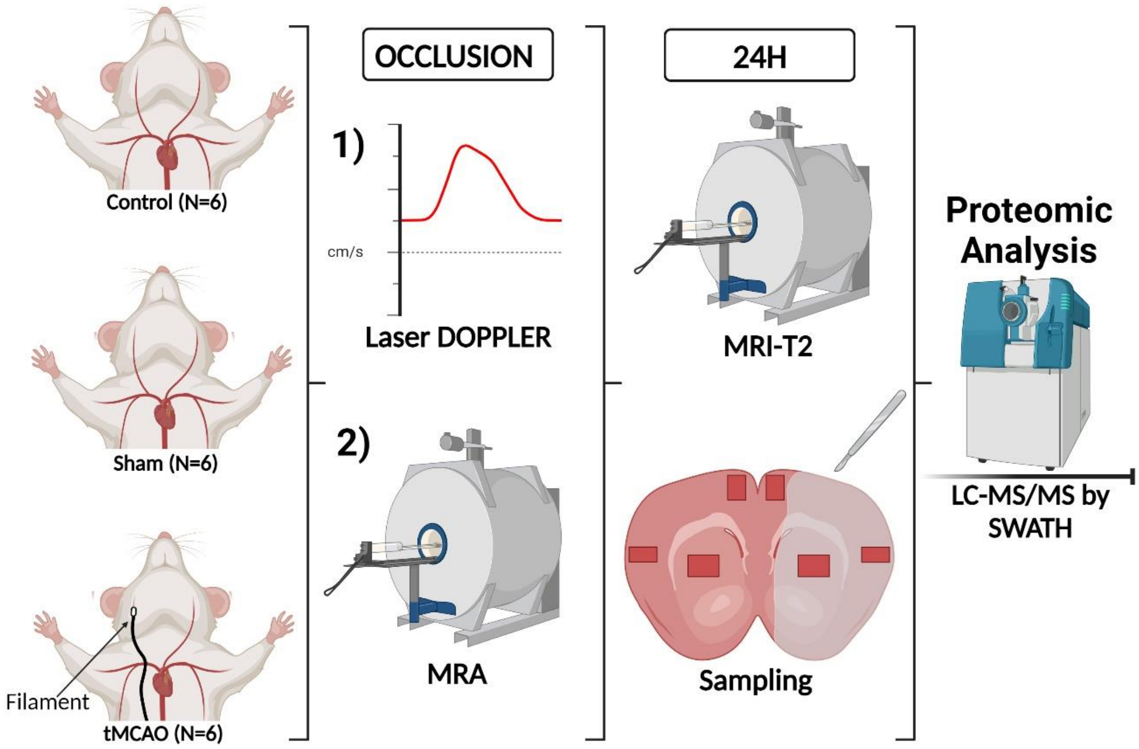

Supplement: Supplementary file 1 [file ijms-25-07538-s001.zip › ijms-3072569-supplementary.pdf]
